# Topics of the Month

**Published:** 1894-08

**Authors:** 


### TOPICS OF THE MONTH.

THE Buffalo Fresh Air Mission Hospital is a charity that should appeal to the kindest sentiments of the human heart. It is open for the reception of young children suffering from acute diseases of the intestinal tract, and contains thirty beds. It is located at Athol Springs, half an hour's ride from Buffalo by either the Lake Shore or the Western New York & Pennsylvania railways.

It would appear to the ordinary observer that the accommodations are inadequate to meet the demands of a great city like Buffalo, and we trust that philanthropic citizens will contribute generously to the enlargement of the hospital as necessity arises.

Dr. Irving M. Snow and Dr. Dewitt H. Sherman, of Buffalo, are the attending physicians. A house physician is on duty at the hospital and trained nurses attend upon the little patients. All such cases should be sent at once to the country where the advantages of fresh air as well as methodical treatment can be invoked to save life.

There is no charge for the care of the infant or its mother. As it is especially desired that the little patients shall be sent at once into the country, with no delay or vexatious formality for physician or parents or friends, any one wishing to send a patient to the Fresh Air Mission Hospital should go or telephone to the Fitch Institute, or to the residence of Dr. Snow or Dr. Sherman ; or the sick child may be brought to the University Dispensary, 24 High street, between 4 and 5 P. M., or to the Fitch Dispensary, corner Swan and Michigan streets, between 10 and 11 A. M. Information and tickets will be furnished at these places.

---

THE board of managers of the State Industrial School at Rochester has transmitted its forty-fifth annual report to the legislature, which covers the workings of the school for the year 1893. We are indebted to Dr. B. L. Hovey, one of the board of managers, for a copy of this interesting brochure.

This institution was formerly known as the Western House of Refuge, and for a number of years was conducted largely on the prison plan. Under the present system, however, it has become an educational institution of great import, including literary and manual training. The contract system has been abolished, trade schools established, and the introduction of military training has added an important part to the machinery of the school. The experts who passed judgment upon the work of this institution at the Chicago exposition last year, framed their award as follows: "For excellence of system, thorough teaching of the trades, careful and judicious management of pupils. The aim of the institution is to reform rather than to punish."

This report is handsomely printed on heavy book paper and is copiously illustrated with interior and exterior views of the institution and its inmates. The composition, press-work and binding of the report are the work of the boys employed in the printing office attached to the school.

---

THERE has always been an impression among a certain portion of the newspaper fraternity that physicians must advertise. The *National Advertiser* has lately taken occasion to say that the predominating feature of the worn and antiquate code of ethics of the American Medical Association is that physicians under all circumstances must obtain all the free advertising possible, and in no case pay for an advertisement under the penalty of being cast aside as a quack. After evolving this brilliant idea, this erudite editor proceeds to ask with all the wisdom of a sophomore: "How long does the press of the country intend to allow itself to be used as a tool by the followers of this ancient code?" The *Kansas Medical Journal* answers this question in a most satisfactory manner. The editor proceeds to remark that there are some points in the code that he thinks should be expunged, but the section relative to advertising is not one of them. He very truthfully asserts that a physician has nothing to advertise, and after discussing the matter at some length he answers those newspapers that have more or less fondness for raising questions similar to that quoted from the *Advertiser*, as follows:

A newspaper which advertises a quack nostrum guaranteed to cure all the ills that man is heir to, destroys confidence in its legitimate advertisements and lowers its value as an advertising medium.

As long as the columns of all the daily and religious newspapers are filled with advertisements of quack nostrums, cure-alls, and medicines to prevent conception and produce abortion, physicians can hardly feel that these are legitimate mediums for reputable advertising.

---

THE assassination of M. Carnot, the able and patriotic president of the republic of France, at Lyon, June 24, 1894, startled the world and produced a profound sensation among its governments. The death wound was one of rare occurrence, caused by a stab in the liver, while President Carnot was receiving the plaudits of his countrymen on his way to the theatre in performance of his function relating to the exposition. He almost immediately sank in collapse and died in a few hours. It was found at the autopsy that the wound was seated in the right side, three centimeters below the xyphoid cartilage. It measured from twenty to twenty-five millimeters, and the blade had completely divided the corresponding costal cartilage. The blade of the knife penetrated the left lobe of the liver, perforating the organ about the suspensory ligaments from left to right and from above downward, in its passage wounding the vena porta, which was opened in two places. The length of the wound in the interior of the liver was eleven to twelve centimeters, and a fatal intraperitoneal hemorrhage was the result of this double perforation. Abdominal section was use less in such a condition, as the fatal nature of the wound was evident from the outset. The only consolation from such a foul thrust is that the agony was soon ended. The judgment of the assassin should be swift and terrible.

---

THE great railroad strike that began at Chicago in the last days of June and spread rapidly throughout the West, finally reaching the Golden Gate, has at last subsided, and business traffic is resuming its normal status.

As medical journalists we have no occasion to discuss the causes that led to this unfortunate and extensive disruption of the relations of labor to capital, but the results thereof afford a legitimate field for comment. The loss of human life resultant therefrom is something terrible to contemplate; while the maimed, whose numbers will probably never be absolutely known, deserve that sympathy that belongs to unfortunate though ignorant and misguided people.

In view of all this suffering, it is a pleasant reflection to know that the medical profession, always humane and charitable, performed its whole duty towards alleviating the sufferings of the poor victims of the folly of Debs and his associates, but it is a pity that the general fund of the American Railway Union cannot be made to open its coffers and pay every dollar of damage, including adequate compensation to the surgeons who ministered to the wounded and dying.

The military under command of Major-General Nelson A. Miles, U. S. A., who, by the way, will soon be commander-in-chief of the army, performed its delicate duty in a masterful manner. The propriety was demonstrated in this strike of establishing government control through the aid of the army with the utmost promptness, and we hope hereafter in all similar outbreaks the precedent established by the government in the Chicago strike will be followed. This is demanded equally on grounds of humanity and economics.

---

WE PUBLISH on another page in this issue of the JOURNAL an interesting article from the pen of William Pryor Letchworth, Esq., commissioner and formerly president of the New York State Board of Charities, entitled, Provision for Epileptics.

Mr. Letchworth is a well-known writer on charity economics, and his present paper will command attention everywhere by reason of the importance of the subject and the interesting manner in which he deals with it.

The interest in epileptics is everywhere increasing, and it will not be long before all the states will be compelled by public opinion to follow the example of Ohio and New York in establishing colonies for their care. We commend Mr. Letchworth's article to the careful perusal of every intelligent physician.

---

THE old-fashioned county medical fee-bill is becoming obsolete. It is a question whether it does any good or not, with the probabilities strongly in the direction that it is not in any way beneficial to the profession. A physician is quite apt to demand and obtain from his patients the value that he himself places on his services, and this whether his county medical society has a standard fee-bill or not.

It used to be thought that a fee-bill prevented the acceptance of remuneration that was on all hands regarded as dishonorably low, but experience has proven that this is not the case.

Again, the legality of the fee-bill is questionable. In Kansas a physician began suit for his fee, and in support thereof a fee-bill was introduced in evidence to show that his charge was not exorbitant, but he lost his case.

